# Calorie Restriction Attenuates Terminal Differentiation of Immune Cells

**DOI:** 10.3389/fimmu.2016.00667

**Published:** 2017-01-12

**Authors:** Matthew J. White, Charlotte M. Beaver, Martin R. Goodier, Christian Bottomley, Carolyn M. Nielsen, Asia-Sophia F. M. Wolf, Luisa Boldrin, Charlotte Whitmore, Jennifer Morgan, Daniel J. Pearce, Eleanor M. Riley

**Affiliations:** ^1^Department of Immunology and Infection, London School of Hygiene and Tropical Medicine, London, UK; ^2^UCL Institute of Healthy Ageing, University College London, London, UK; ^3^Department of Infectious Disease Epidemiology, London School of Hygiene and Tropical Medicine, London, UK; ^4^Dubowitz Neuromuscular Centre, Developmental Neurosciences Programme, Molecular Neurosciences Section, Institute of Child Health, University College London, London, UK

**Keywords:** natural killer cell, T cell, calorie restriction, aging, differentiation, maturation

## Abstract

Immune senescence is a natural consequence of aging and may contribute to frailty and loss of homeostasis in later life. Calorie restriction increases healthy life-span in C57BL/6J (but not DBA/2J) mice, but whether this is related to preservation of immune function, and how it interacts with aging, is unclear. We compared phenotypic and functional characteristics of natural killer (NK) cells and T cells, across the lifespan, of calorie-restricted (CR) and control C57BL/6 and DBA/2 mice. Calorie restriction preserves a naïve T cell phenotype and an immature NK cell phenotype as mice age. The splenic T cell populations of CR mice had higher proportions of CD11a^−^CD44^lo^ cells, lower expression of TRAIL, KLRG1, and CXCR3, and higher expression of CD127, compared to control mice. Similarly, splenic NK cells from CR mice had higher proportions of less differentiated CD11b^−^CD27^+^ cells and correspondingly lower proportions of highly differentiated CD11b^+^CD27^−^NK cells. Within each of these subsets, cells from CR mice had higher expression of CD127, CD25, TRAIL, NKG2A/C/E, and CXCR3 and lower expression of KLRG1 and Ly49 receptors compared to controls. The effects of calorie restriction on lymphoid cell populations in lung, liver, and lymph nodes were identical to those seen in the spleen, indicating that this is a system-wide effect. The impact of calorie restriction on NK cell and T cell maturation is much more profound than the effect of aging and, indeed, calorie restriction attenuates these age-associated changes. Importantly, the effects of calorie restriction on lymphocyte maturation were more marked in C57BL/6 than in DBA/2J mice indicating that delayed lymphocyte maturation correlates with extended lifespan. These findings have implications for understanding the interaction between nutritional status, immunity, and healthy lifespan in aging populations.

## Introduction

Obesity, diet, and our aging population are increasingly important public health concerns in many societies (1). Whereas excess calorie intake has been linked to increased incidence of many chronic degenerative diseases (2, 3), calorie restriction reduces blood triglyceride concentrations, reduces the incidence of hypertension, heart disease, kidney disease, and neurological dysfunction, increases sensitivity to insulin, and is associated with increased longevity (4–11). However, it is difficult to quantify the benefits of calorie restriction *per se* in human populations, or to evaluate how calorie restriction interacts with age, since voluntary calorie restriction is often associated with other healthier life-style choices that can confound interpretations (12, 13).

In mice, calorie restriction enhances responses to vaccination, reduces the incidence of spontaneous malignancies, and, in some inbred strains, extends lifespan (14, 15). Specifically, restriction of the calorie intake of C57BL/6J mice by 40% compared to that of mice fed *ad libitum* (AL), extends median lifespan by more than 35% (i.e., from around 24 months to around 32 months) whereas the lifespan of DBA/2J mice is not extended by calorie restriction (16–18). This differential response to calorie restriction may be linked to lower basal metabolic rate, lower oxygen consumption, higher oxidative stress, higher body fat, and continued weight gain throughout adult life in C57BL/6 mice compared to DBA/2 mice fed AL (18, 19) although differential effects on nutrient sensing cannot be ruled out (20, 21).

Importantly, age-associated changes in the adaptive immune system—typified by thymic involution, reduced production of naïve T cells, reduced T cell proliferation, reduced cytotoxic T lymphocyte activity, and progressive skewing of the T cell pool toward more mature, memory phenotypes with increasing age (22)—are attenuated by calorie restriction. In mice and in non-human primates, calorie restriction conserves T cell function and repertoire and promotes production and/or maintenance of naïve T cells (22). The effects of aging and calorie restriction on the innate immune system are, however, much less well studied. Altered function of innate cell lineages of aged individuals (23) has been linked to defective immune regulation and chronic inflammation (24–28). In particular, age-associated dysfunction of natural killer (NK) cells has been reported in mice (29, 30) and humans (31).

Natural killer cells are large granular lymphocytes that contribute to both innate and adaptive immune responses by direct lysis of malignant, stressed or virally infected cells, by cytokine production, and by antibody-dependent cellular cytotoxicity (ADCC) (32). The diverse functions of NK cells are dictated in part by their differentiation state. In humans, down regulation of CD56 (CD56^bright^ to CD56^dim^) followed by expression of CD57 (CD57^−^ to CD57^intermediate^ to CD57^+^) marks the stepwise differentiation of NK cells from cytokine-responsive and cytokine-secreting cells toward cells specialized in ADCC (33–38). CD56^dim^ CD57^+^ NK cells accumulate gradually with increasing age and this process is accelerated in human cytomegalovirus infected individuals (39, 40). Progressive narrowing of the NK cell functional repertoire with increasing age may contribute to immune senescence (26).

In mice, stepwise differentiation of NK cells (defined as NKp46^+^ NK1.1^+^ CD3^−^ lymphocytes) is characterized by loss of CD27 expression and gain of CD11b (41). Peripheral NK cell numbers fall in aged mice (30) but—in contrast to what is seen for T cells [i.e., accumulation of memory cells and terminally differentiated effectors (22)]—this is associated with loss of the most mature NK cell subset (CD27^−^ CD11b^+^) in aged animals (30). Moreover, NK cells in aged mice appear functionally impaired *in vivo* (e.g., in response to influenza virus) and *in vitro* (e.g., in response to cytokines, MHC class I deficient target cells or receptor cross-linking) (29, 30, 42, 43). Calorie restriction seems to mimic the effects of aging on murine NK cells, with 40% calorie restriction leading to reduced numbers of peripheral NK cells and decreased proportions of the most differentiated NK cell subset in 6-month-old C57BL/6 mice (44). NK cells from these calorie-restricted (CR) mice expressed higher levels of T-bet, Eomes, CD127 (IL-7Rα), and CD27 and produced TNF and GM-CSF but had correspondingly decreased expression of KLRG1, CD11b, and Ly49 receptors and lower IFN-γ production and degranulation (44). One study also suggested that CR mice are more susceptible to influenza A infection, with increased viral titers and lower NK cell activity *in vivo* and *in vitro* (45), again mimicking the effects of aging (29), although the causal relationship between NK cell function and outcome of infection remains to be tested.

Despite evidence that calorie-restriction appears to mimic the effects of aging in murine NK cells, calorie restriction enhances healthy life span in C57BL/6 mice (17) suggesting that age-related changes in murine NK cells may have evolved to preserve innate immune function, and thus resilience in the face of infection, in adult life and thus that there is an underlying unappreciated interaction between age and calorie intake. In an attempt to reveal this interaction, we have—for the first time—analyzed the effects of calorie restriction on NK cell and T cell phenotype and function throughout the life course in C57BL/6J and DBA/2J mice.

## Materials and Methods

### Experimental Animals

Twelve-week-old male and female C57Bl/6J and DBA/2J mice were purchased from Charles River Laboratories. One week after arrival, mice were weighed and individually housed. The AL group was given unrestricted access to 18% Protein Rodent Diet (Harlan, UK) and their average daily food consumption was calculated each week. At 14 weeks of age, the CR group was provided with 90% of the average daily food consumption of the AL group for the previous week; this was reduced to 75% the following week and then to 60% for the remainder of the study. Average food consumption per week of AL animals ranged from 23.7 to 31.8 g. AL DBA/2 mice typically consumed 7–19% more food per week than age matched C57Bl/6 mice. Animals were weighed every 4 weeks and any animal that lost 20% of its weight over that period, or showed other signs of ill health, was removed from the study and humanely killed. Between 10 and 13 mice (approximately equal numbers of male and female) from each group and of each strain were humanely killed at each time point (6, 12, and 22 months) (Figure S1 in Supplementary Material). Overall, 88% of mice survived to their scheduled endpoint. Of those that were humanely killed prior to the anticipated endpoint, the majority (77%) were in the 22-month-old study group and 64% were in the CR groups; 76% of mice in the 22-month-old study groups survived until the end of the experiment. All procedures were performed in accordance with the United Kingdom Animals (Scientific Procedures) Act 1986 and University College London institutional guidelines and with consent and monitoring by the local Animal Welfare and Ethical Review Body (AWERB).

### Spleen Cell Preparation

Lymphocytes were isolated from spleen, liver, lung, and lymph nodes by mechanical disruption of individual organs through a 70-µm cell strainer into complete RMPI [RPMI-1640 supplemented with 100 U/ml penicillin/streptomycin, 20 mM l-glutamine (Gibco, Lifesciences, Paisley, UK), and 1% fetal bovine serum (Sigma, UK)]. Contaminating red blood cells were lysed with Pharm Lyse buffer (BD Biosciences, Oxford, UK). Cells were then washed twice more in complete medium, resuspended in freezing medium [90% FBS, 10% DMSO (Sigma, UK)], and cryopreserved in liquid nitrogen. Prior to use, cells were thawed in pre-warmed complete medium (at 37°C), washed three times, counted, and rested for 30 min before use. Cell viability by trypan blue exclusion was >99%.

### Cell Culture

Thawed, washed, and rested lymphocytes were cultured in 96-well U-bottom plates, at a concentration of 2 × 10^5^ cells in a total volume of 200 µl of complete medium, for 18 h (37°C, 5% CO_2_) with or without IL-12 plus IL-18 [1 ng/ml recombinant mouse (rm)-IL-12 (PeproTech, Rocky Hill, NJ, USA) plus 20 ng/ml rmIL-18 (MBL, Woburn, MA, USA)] or IL-2 [100 ng/ml rmIL-2 (PeproTech)]. Two microliters of anti-CD107a-FITC were added to each well after 14 h and GolgiPlug (containing Brefeldin A, 1/1,000 final concentration; BD biosciences, Oxford, UK) and GolgiStop (containing Monensin, 1/1,500 concentration; BD biosciences, Oxford, UK) were added after 15 h.

### Flow Cytometry

Natural killer cell and T cell phenotypes were assessed both *ex vivo* and after *in vitro* culture by flow cytometry. Briefly, up to 5 × 10^5^ cells were incubated with fluorophore-labeled monoclonal antibodies to cell surface molecules, fixed and permeabilized [Cytofix/Cytoperm (BD Biosciences) or FoxP3 fix/perm (e-Bioscience/Affymetrix, Hatfield, UK)], and stained for intracellular molecules. Cells were incubated with combinations of the following rat-anti-mouse antibodies: anti-CD4-V500, anti-CD11b-V500, anti-CD107a-FITC, and anti-CD69-PerCP-Cy5.5 (all from BD Biosciences); anti-CD127-FITC, anti-NKG2A/C/E-FITC, anti-FoxP3-FITC, anti-Ly49G2-FITC, anti-Ly49H-FITC, anti-NKp46-(PE or e450), anti-TRAIL-PE, anti-TCRβ-PE, anti-LY49C/I/F/H-PE, anti-Ly49A/D-PE, anti-CD3-(PerCP-Cy5.5 or e450 or allophycocyanin-e780), anti-CD8-PE-Cy5, anti-KLRG1-PE-Cy7, anti-CD44-PE-Cy7, anti-CD27-(PE-Cy7 or allophycocyanin-e780), anti-CD218a-e450, anti-CXCR3-allophycocyanin, anti-CD11a-allophycocyanin, anti-CD25-(allophycocyanin or allophycocyanin-e780), anti-Ly49E/F-allophycocyanin, anti-Ly49D-allophycocyanin, anti-Ki67-allophycocyanin, and anti-IFNγ-allophycocyanin-e780 (all from eBioscience). All antibodies stained cells from both C57BL/6 mice and DBA/2 mice with the exception of the anti-NKG2A/C/E antibody and four of the Ly49 receptor antibodies (Ly49A, D, H and F), all of which failed to stain cells from DBA mice, which could be due to either very low surface expression or allelic differences not recognized by the specific monoclonal antibody (46). Cells were analyzed by LSR II (BD Biosciences). Acquired events were gated as single cells. Due to channel limitations and as cell viability was >99% by trypan blue exclusion, a live-dead marker was not used but dead cells were excluded on the basis of size and side scatter.

### Data Analysis

Flow cytometry data were analyzed using Flow Jo (Tree Star) and exported into Prism6 (GraphPad, San Diego, CA, USA). Any sample with fewer than 100 cells in any particular subset was excluded from the analysis of that subset. The Mann Whitney *U* test was used to compare the CR and control groups and one-way analysis of variance (ANOVA) was used to compare the age groups. We tested for differences between the CR and control groups in the association with age by including an interaction term in a two-way ANOVA. Data are represented by box and whisker plots showing median, interquartile range (box), and range (bars) with individual data points shown. Significant differences between AL and CR groups are shown above the data. Significant differences between strains are indicated in the figure legends, where appropriate: ****p ≤ 0.0001, ***p < 0.001, **p < 0.01, and *p < 0.05.

## Results

From the age of 16 weeks, approximately equal numbers of male and female C57/BL6 and DBA/2 mice were fed either AL or CR by being fed 60% of the average daily food consumption of the AL group for the previous week. Each group comprised between 8 and 13 mice. Groups of mice of each strain were humanely killed at 6, 12, and 22 months. Body weights of control (AL) C57BL/6 mice increased over the entire life course whereas weights of AL DBA mice stabilized at approximately 12 months of age (Figure S2 in Supplementary Material). Body weights of CR mice were significantly lower than their respective AL controls at all time points in both strains, with the size of this difference increasing with duration of calorie restriction and thus with age (Figure S2 in Supplementary Material). Among CR mice, there was no significant increase in body weight with age among C57BL/6 mice and the average weight of CR DBA/2 mice decreased significantly with age. Among the control (AL) C57BL/6 mice, males weighed significantly more than females at all ages, but this difference was much less marked among the CR mice (Figures S2B,C in Supplementary Material). Among the DBA/2 mice, there was no significant difference in weights of male and female CR mice at 12 and 22 months of age (Figure S2C in Supplementary Material).

Lymphocyte populations from spleen, lung, liver, and lymph nodes were analyzed. Observations from lung, liver, and lymph nodes were essentially identical to those from spleen cells and thus only spleen cell data are presented. Spleens from CR mice were visibly smaller than those from control (AL) animals (data not shown) but this did not translate into lower spleen cell counts (e.g., Figure S2D in Supplementary Material).

### Age-Related Changes in T Cell Phenotype Are Delayed by Calorie Restriction

**Figure 1:**
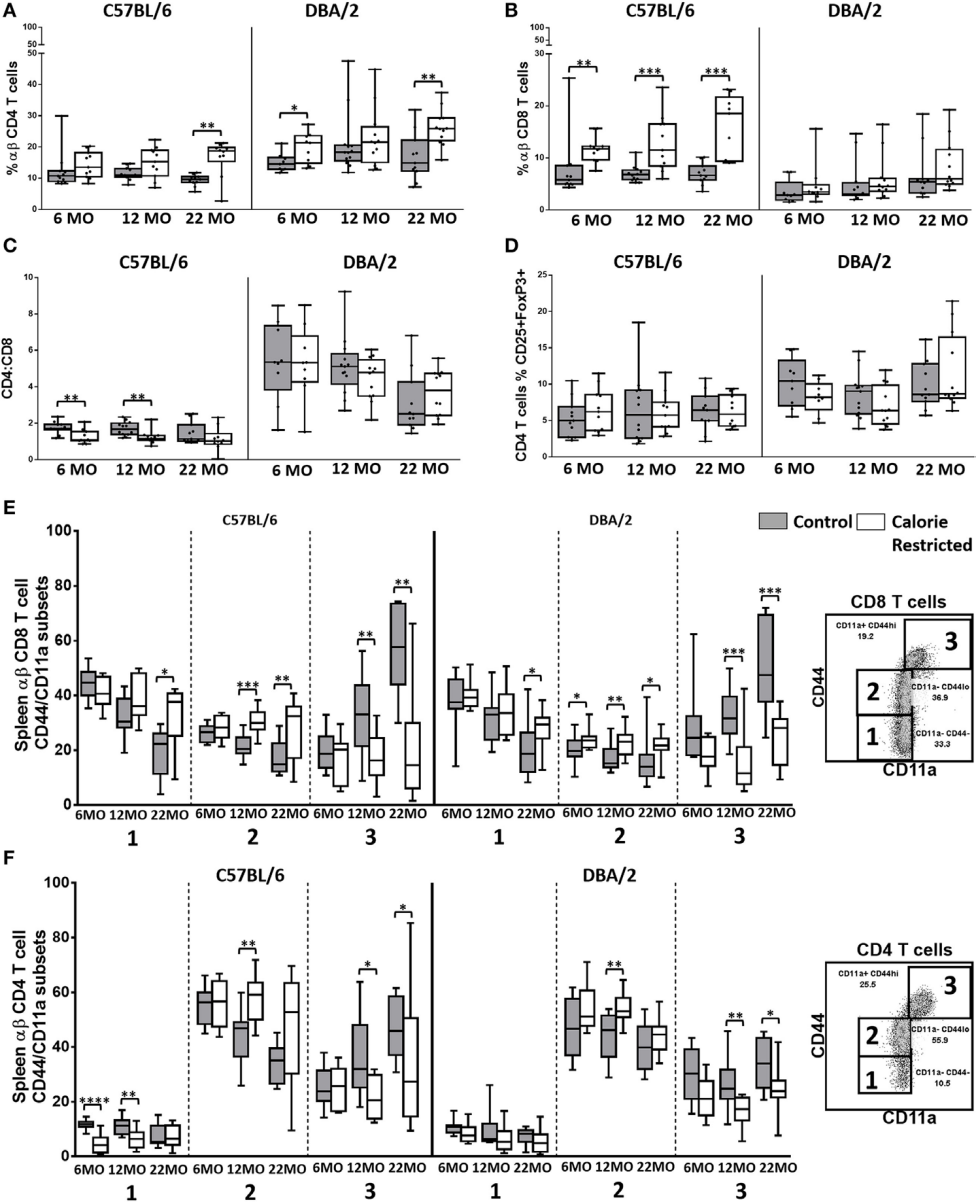
**T cell frequencies and activation state are altered by calorie-restricted (CR) and aging**. Characterization of murine splenic T cells in control (shaded boxes) and CR mice (open boxes) at 6 months (6 MO), 12 months (12 MO), and 22 months (22 MO) of age in C57BL/6J and DBA/2J mice. Significant differences between strains are shown in parentheses. T cells were gated as CD3^+^, TCRβ^+^ CD4^+^, or CD8^+^ (for gating, see Figure S3A in Supplementary Material). Plots represent percentage of all lymphocytes that are **(A)** CD4^+^ T cells (6 MO C**, CR*; 12 MO C****, CR*; 22 MO C**, CR****) or **(B)** CD8^+^ T cells (6 MO C**, CR***; 12 MO C**, CR***; 22 MO CR**); **(C)** CD4:CD8 ratio (6 MO C**, CR****; 12 MO C****, CR****; 22 MO C**, CR****); **(D)** percentage of CD4 T cells that are FoxP3^+^ CD25^+^ T_regs_ (6 MO C**; 22 MO C*, CR**); and percentage of CD8^+^ T cells **(E)** or CD4^+^ T cells **(F)** that belong to each of the CD44/CD11a defined subsets, as defined in the embedded flow cytometry plots. Horizontal bars represent medians, boxes extend from the 25th to the 75th percentile, and whiskers represent the min to max range. *p* Values are derived from non-parametric Mann Whitney *U* tests. ****p ≤ 0.0001, ***p < 0.001, **p < 0.01, and *p < 0.05. Numbers of mice per group ranged from 10 to 13.

**Figure 2:**
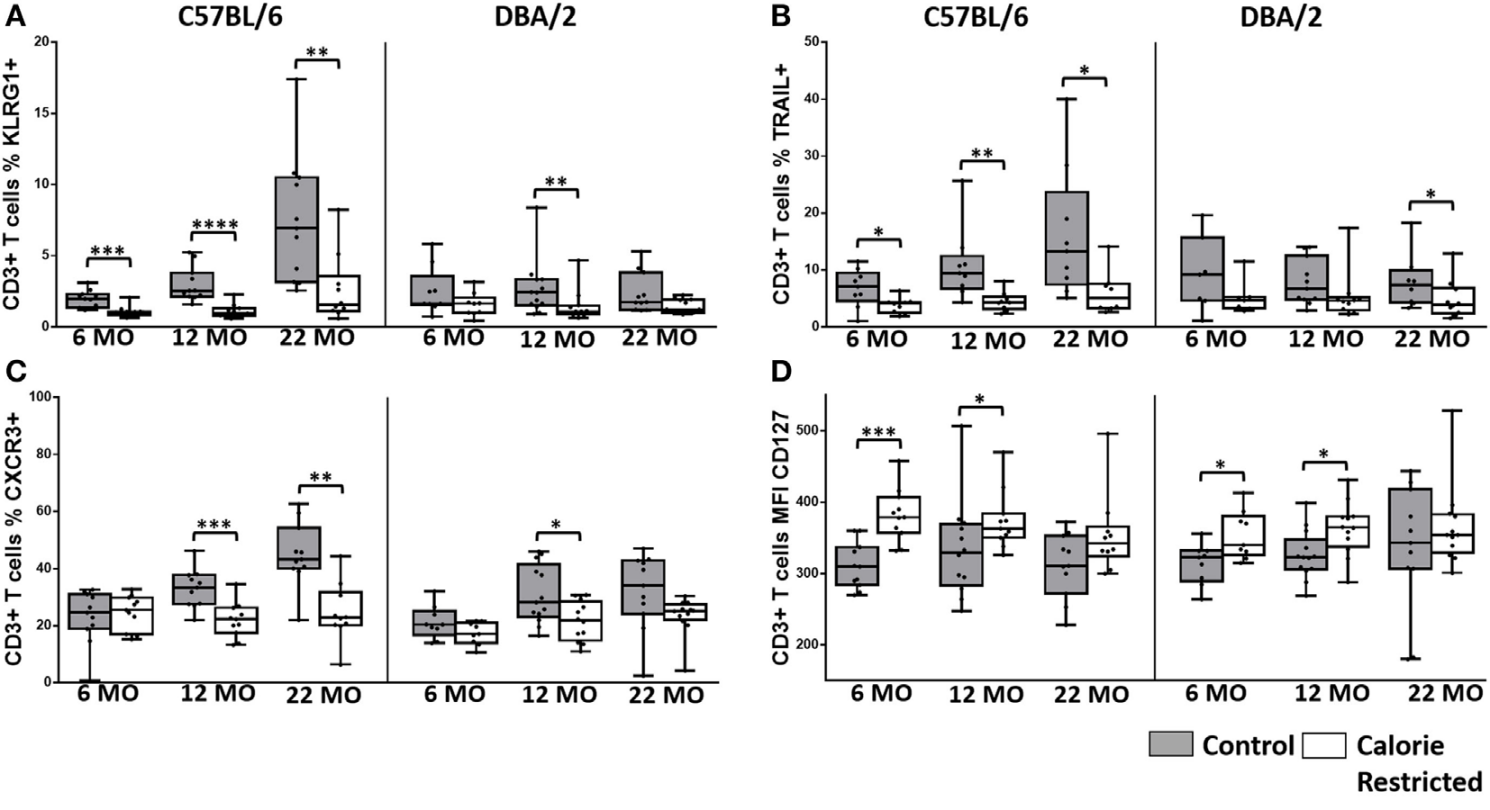
**T cell phenotype is altered by calorie-restricted (CR) and age**. Characterization of murine splenic T cells in control (shaded boxes) and CR mice (open boxes) at 6 months (6 MO), 12 months (12 MO), and 22 months (22 MO) of age in C57BL/6J and DBA/2J mice. Significant differences between strains are shown in parentheses. T cells were gated as CD3^+^ NKp46^−^ (for gating, see Figure S3B in Supplementary Material). Plots represent the percentage of all T cells expressing **(A)** KLRG1 (22 MO C*), **(B)** TRAIL and **(C)** CXCR3 (6 MO CR*; 22 MO C*), and **(D)** the median fluorescence intensity of CD127 expression by T cells. Horizontal bars represent median values, boxes extend from the 25th to the 75th percentile, and whiskers represent the min to max range. *p* Values are derived from non-parametric Mann Whitney *U* tests. ****p ≤ 0.0001, ***p < 0.001, **p < 0.01, and *p < 0.05. Numbers of mice per group ranged from 10 to 13.

The effects of aging on T cells are well described (22) but the effects of CR are much less well studied. Although limited by cell numbers, we were able to assess how proportions of αβTCR^+^ CD4^+^ and CD8^+^ T cells and regulatory T cells (CD4^+^ FoxP3^+^ CD25^+^; T_reg_) changed with age and CR (Figure 1) and to evaluate a number of phenotypic markers on these populations (Figures 1 and 2). The gating strategy is shown in Figure S3 in Supplementary Material.

There were no major age-related changes in proportions of CD4^+^, CD8^+^, or T_reg_ among the C57BL/6 mice although subtle but statistically significant increases in percentages of CD8^+^ T cells with age (*p* = 0.01) among DBA/2 mice led to a gradual and significant decline in CD4/CD8 ratio (*p* < 0.001) with increasing age (Figures 1A–D). CD4/CD8 ratios were markedly higher among DBA/2 mice than C57BL/6 mice at all time points in both control and CR groups. Calorie restriction resulted in significantly increased proportions of CD8^+^ T cells in C57BL/6 mice with a corresponding decline in the CD4/CD8 ratio (Figures 1B,C). However, this effect was not seen in DBA/2 mice and there was no significant effect of CR on the proportion of T_regs_ in either strain (Figure 1D).

Proportions of naïve CD4^+^ and CD8^+^ T cells decline with increasing age in mice and humans and this is due, in part, to thymic involution (47, 48): this leads to gradual and progressive skewing of the T cell population toward memory T cell phenotypes (22). In line with these expectations, proportions of CD44^−^/CD11a^−^ (naïve) and CD44^lo^/CD11a^−^ (antigen experienced) CD8^+^ T cells (49, 50) declined with age in C57BL/6 control mice and there was a corresponding increase in proportions of CD44^hi^/CD11a^+^ CD8^+^ effector T cells (49) with age (Figure 1E) (*p* < 0.001, 0.05, and 0.001, respectively). Among control DBA/2 mice, proportions of CD44^−^/CD11a^−^ (naïve) declined with age (*p* < 0.001) and proportions of CD44^hi^/CD11a^+^ CD8^+^ effector T cells increased with age (*p* < 0.001) but proportions of CD44^lo^/CD11a^−^ (antigen experienced) CD8^+^ T cells did not change with age (Figure 1E). Similar effects were seen for CD4^+^ T cells with age-related declines in proportions of CD44^lo^/CD11a^−^ cells and a corresponding increase in CD44^hi^/CD11a^+^ CD4^+^ T cells (*p* < 0.001 in all cases) (Figure 1F). Among C57BL/6 mice, this shift from naïve to effector T cell phenotype was markedly delayed in CR mice, with the naïve/effector T cell distribution of 22-month-old CR mice resembling that of 6-month-old control animals (interaction term for age and diet, *p* < 0.05 in all cases) but no significant interaction was seen between age and diet in DBA/2 mice (Figures 1E,F).

To further investigate the effects of calorie restriction on T cell phenotype, CD3^+^ T cells were assessed for their expression of KLRG1, TRAIL, CXCR3, and CD127; limiting cell numbers precluded analysis by CD4^+^ or CD8^+^ status (for gating, see Figure S3B in Supplementary Material).

Consistent with use of KLRG1 as a marker of senescence and loss of function in antigen experienced T cells (51), we found that CR markedly delayed the age-associated appearance of KLRG1^+^ T cells (Figure 2A). This effect was, again, most pronounced in C57BL/6 mice although this was due in part to very low levels of KLRG1 expression on DBA/2 T cells at all ages, irrespective of nutritional status. In addition, there was a significant interaction between diet and age in C57BL/6 mice (*p* = 0.01) but not in DBA/2 mice (*p* = 0.9). Very similar trends were seen for TRAIL expression (Figure 2B), although in this case, there was no significant interaction between age and diet in either strain. CXCR3 is rapidly acquired on activated T cells and is maintained on effector T cells (52). The proportion of CXCR3^+^ T cells increased with age in C57BL/6 mice (*p* < 0.001), expression of CXCR3 was attenuated in CR mice (Figure 2C), and there was a significant interaction between diet and age (*p* < 0.001). Again the effect of CR was much less pronounced in DBA/2 mice and there were no significant age-associated changes in CXCR3 expression in this strain. By contrast, levels of expression (mean fluorescence intensity) of CD127 (IL-7Rα) were maintained with increasing age and were increased by CR, especially in younger mice (Figure 2D). As IL-7 signaling is required for peripheral maintenance of T cells (53) and is lost on terminally differentiated cells (20), these data are consistent with delayed accumulation of terminally differentiated T cells in CR mice.

### Calorie Restriction Exaggerates Age-Associated Changes in NK Cell Phenotype

To our surprise, the effect of calorie restriction on the NK cell population was to exaggerate, rather than attenuate, the normal age-associated changes in NK cell phenotype and function. The gating strategy for NK cell evaluation is shown in Figure S4C in Supplementary Material.

**Figure 3:**
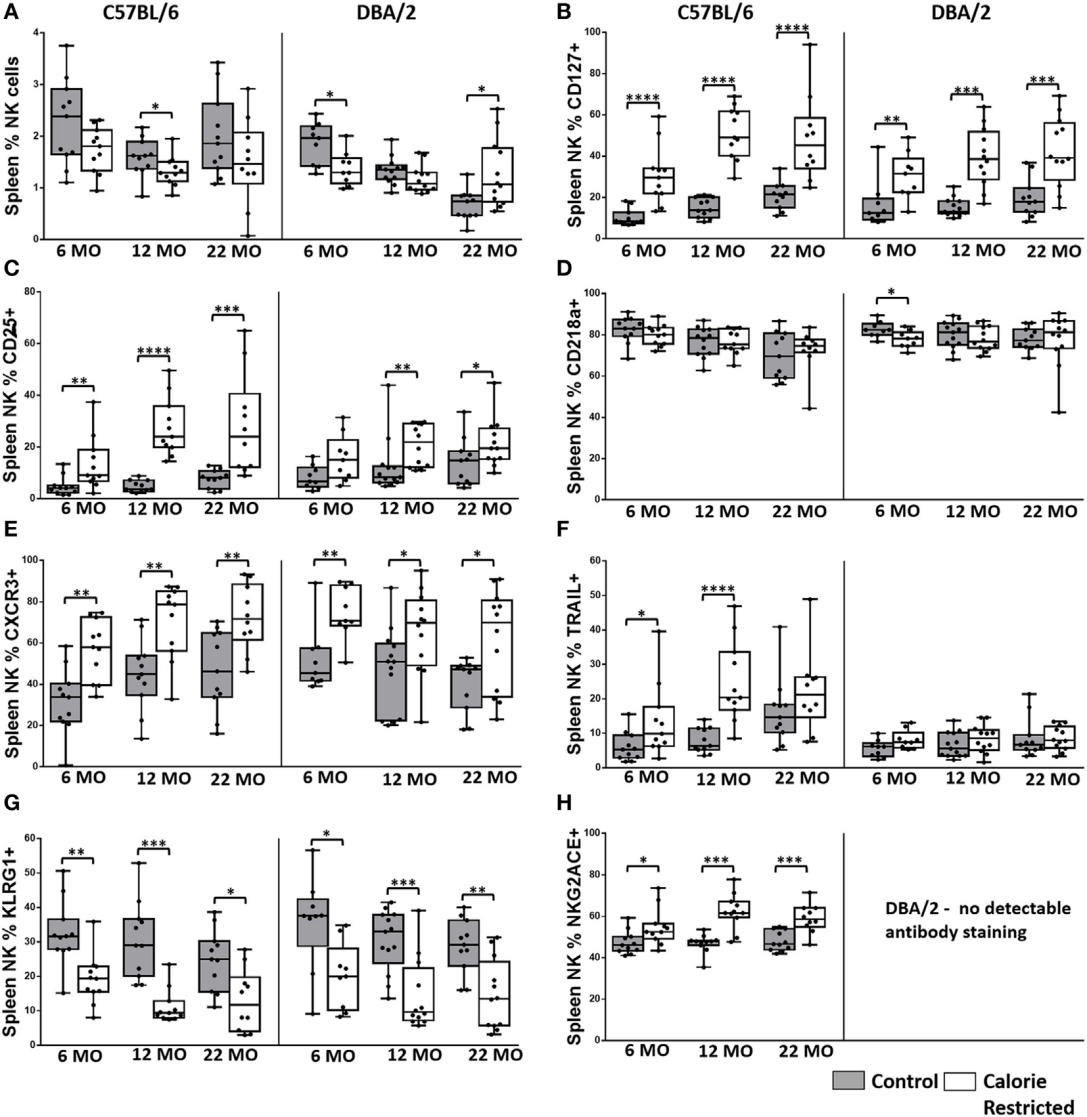
**Natural killer (NK) cell phenotype is altered by calorie-restricted (CR) and age**. Characterization of murine splenic NK cells in control (shaded boxes) and CR mice (open boxes) at 6 months (6 MO), 12 months (12 MO), and 22 months (22 MO) of age in C57BL/6J and DBA/2J mice. Significant differences between strains are shown in parentheses. Plots represent **(A)** percentage of splenic lymphocytes that are NK cells (NKp46^+^ CD3^−^) (12 MO C*; 22 MO C****), or **(B–H)** percentage of NK cells expressing **(B)** CD127 (6 MO C*; 12 MO C*), **(C)** CD25 (6 MO C*; 12 MO C*), **(D)** CD218a, **(E)** CXCR3 (6 MO C**, CR*), **(F)** TRAIL (12 MO C****; 22 MO C*, CR***), **(G)** KLRG1, and **(H)** NKG2A/C/E. For gating, see Figure S4 in Supplementary Material. Horizontal bars represent median values, boxes extend from the 25th to the 75th percentile, and whiskers represent the min to max range. *p* Values are derived from non-parametric Mann Whitney *U* tests. ****p ≤ 0.0001, ***p < 0.001, **p < 0.01, and *p < 0.05. Numbers of mice per group ranged from 10 to 13.

The proportion of NK cells among splenic mononuclear cells declined significantly with age in DBA/2 mice (*p* < 0.001) but not in C57BL/6 mice. Although the effects of diet on NK cell proportions were not consistent (and in the absence of data on absolute cell numbers, it would be unwise to over interpret minor statistically significant differences between AL and CR mice in the proportions of spleen cells that are NK cells), there was a highly statistically significant interaction between age and diet in DBA/2 mice (*p* < 0.001) (Figure 3A). In line with previous reports (26, 30, 54), the surface phenotype of resting NK cells changed significantly with age in control C57BL/6 mice but—remarkably—these age-related changes were not seen in control DBA/2 mice (Figure 3). Moreover, the effects of aging on NK cells were exaggerated in CR mice compared to controls in both strains of mice. Thus, the proportions of NK cells expressing IL-7Rα (CD127), the chemokine receptor CXCR3, and TRAIL all increased significantly with age in C57BL/6 mice (*p* = 0.05, *p* = 0.05, and *p* = 0.01, respectively) and were significantly higher in CR mice compared to control mice; similar trends were seen among CR DBA/2 mice but were not statistically significant. Conversely, proportions of NK cells expressing KLRG1 were markedly affected by CR but not by age whereas expression of IL-18Rα (CD218a) declined with age only in C57BL/6 mice (*p* < 0.001) and there was very little effect of CR. This tendency for CR to exacerbate underlying age-associated trends suggests that the underlying processes affecting the NK cell population are the same in both cases, and that the effects of aging and CR on NK cells are synergistic in aged, CR mice. This is in complete contrast to the effect of CR on T cells (Figures 1 and 2), where CR attenuates age-associated effects.

### Reduced Expression of Ly49 Receptors by NK Cells from CR Mice

**Figure 4:**
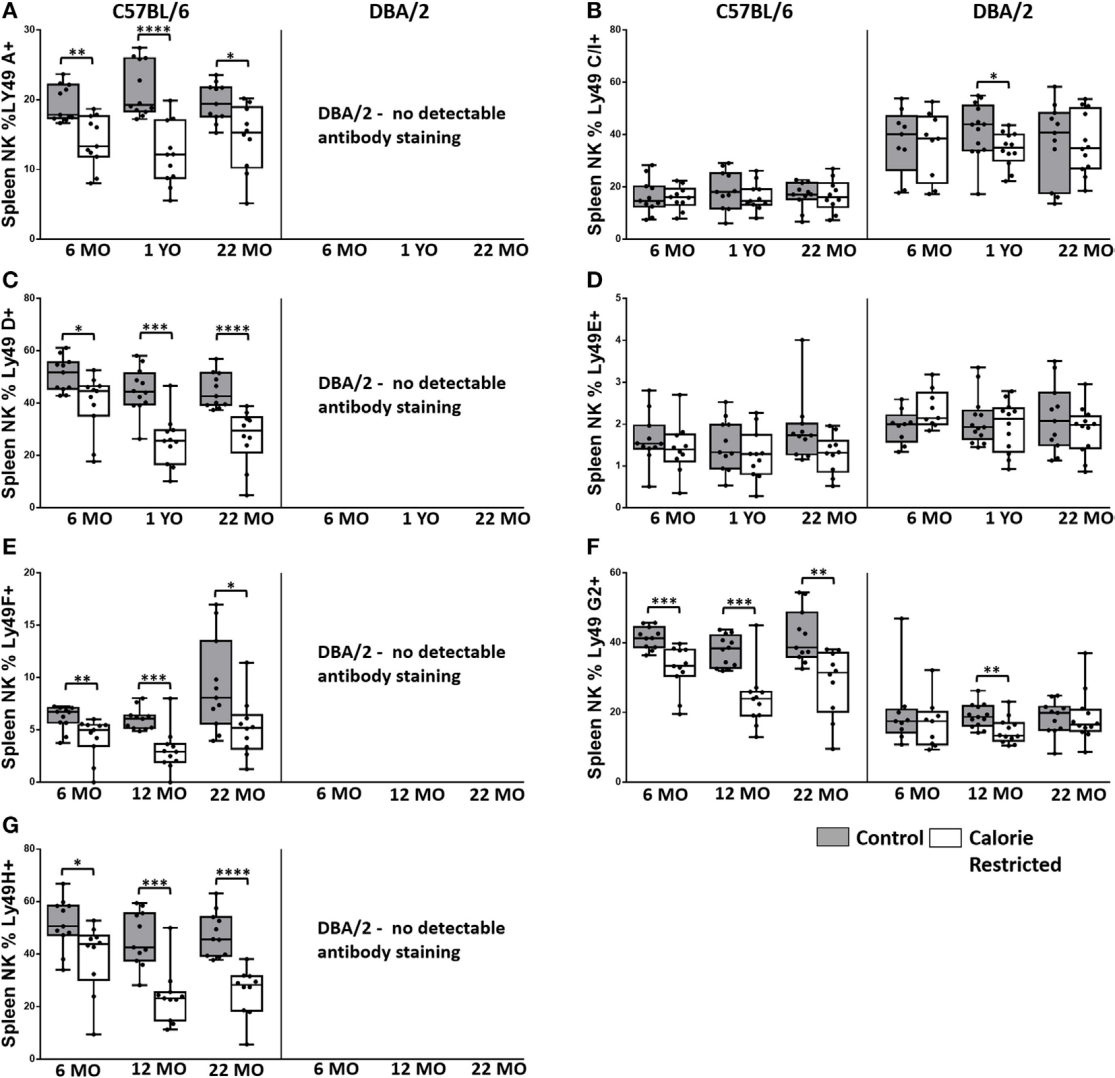
**Ly49R expression changes with calorie-restricted (CR) and age**. Characterization of murine splenic natural killer (NK) cells in control (shaded boxes) and CR mice (open boxes) at 6 months (6 MO), 12 months (12 MO), and 22 months (22 MO) of age in C57BL/6J and DBA/2J mice. Significant differences between strains are shown in parentheses. **(A–G)** Plots represent the percentage NK cells (NKp46^+^, CD3^−^) expressing **(A)** Ly49A, **(B)** Ly49C/I (6 MO C***, CR***; 12 MO C****, CR****; 22 MO C*, CR***), **(C)** Ly49D, **(D)** Ly49E (6 MO CR***; 12 MO CR*; 22 MO CR*), **(E)** Ly49F, **(F)** Ly49G2 (6 MO C**, CR***; 12 MO C****, CR**; 22 MO C****, CR*), and **(G)** Ly49H. For gating, see Figure S4 in Supplementary Material. Horizontal bars represent median values, boxes extend from the 25th to the 75th percentile, and whiskers represent the min to max range. *p* Values are derived from non-parametric Mann Whitney *U* tests. ****p ≤ 0.0001, ***p < 0.001, **p < 0.01, and *p < 0.05. Numbers of mice per group ranged from 10 to 13.

Ly49 receptors are C-type lectin receptors for MHC Class I molecules and regulate altered/missing self-recognition by murine NK cells; most Ly49 receptors transmit inhibitory signals although some mediate NK cell activation (55, 56). We assessed the expression of two activating (Ly49D and Ly49H) and six inhibitory (Ly49A, Ly49C/I, Ly49E, Ly49F, and Ly49G2) receptors in the context of strain, CR, and age (Figure 4). Overall, in C57BL/6 mice, Ly49 receptor was relatively stable with age, although there was a tendency for the proportion of Ly49D^+^ cells to decrease with increasing age in both control and CR mice (*p* = 0.04) and for Ly49F^+^ cells to increase with age (*p* = 0.14). However, in line with reports that Ly49 receptor expression increases as NK cells mature (57) and with the indication (above) that proportions of mature NK cells are decreased in CR mice, proportions of Ly49A^+^, Ly49D^+^, Ly49F^+^, Ly49G2^+^, and Ly49H^+^ NK cells were markedly and significantly lower in CR mice compared to control mice, although no effect of CR was seen for Ly49E or Ly49C/I expressing cells (Figure 4).

Comparison of Ly49 receptor expression between the two strains of mice was complicated by the fact that we could not detect Ly49A, Ly49D, Ly49F, or Ly49H on cells from DBA/2 mice; however, there was no effect of CR on expression of two of the receptors that could be detected in DBA/2 mice (Ly49E and Ly49C/I). Differences in Ly49 expression between C57BL/6 and DBA/2—including higher expression of Ly49 C/I, lower expression of Ly49G2, and no detectable expression of Ly49D and Ly49H mRNA in DBA/2 mice—have been reported previously (46, 58); Ly49A mRNA, but not protein, has been detected in DBA/2 mice (58). This is the first report of lack of detectable Ly49F expression in DBA/2 mice and may reflect either true lack of expression in this strain or polymorphism affecting antibody binding.

### Calorie Restriction Is Associated with Accumulation of Early Stage/Immature NK Cells

**Figure 5:**
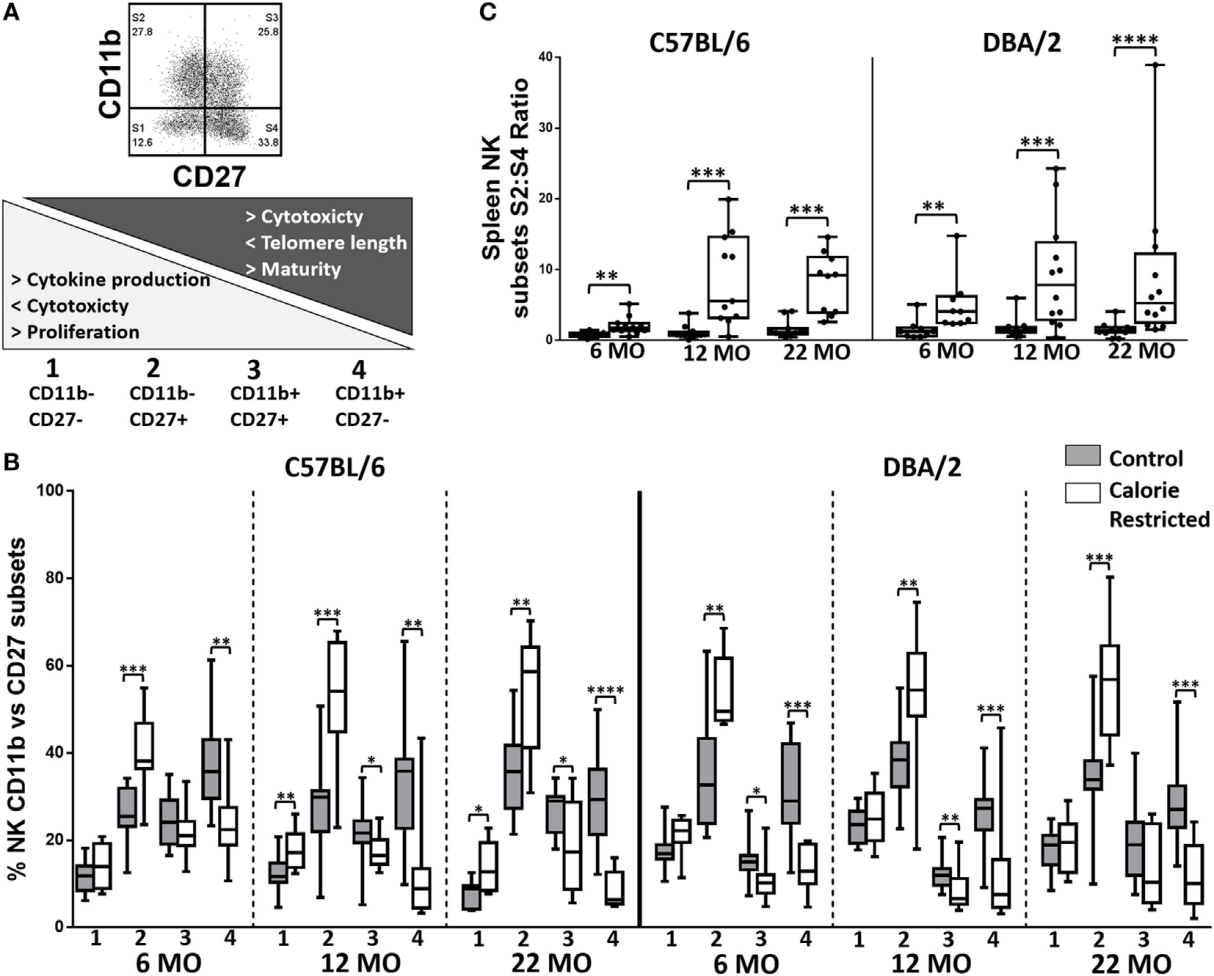
**Natural killer (NK) cell maturation is delayed in calorie-restricted (CR) mice**. Maturation phenotype of murine splenic NK cells in control (shaded boxes) and CR mice (open boxes) at 6 months (6 MO), 12 months (12 MO), and 22 months (22 MO) of age in C57BL/6J and DBA/2J mice. **(A)** Gating and definition of CD27/CD11b defined NK cell subsets, **(B)** percentage of NK cells in each CD11b/CD27 defined subset; the numbers below the *x*-axis refer to the NK cell subsets as defined in **(A)**, **(C)** ratio of S2:S4 NK cell subsets; significant differences between strains were observed for 6 MO CR** and 12 MO C* mice. Horizontal bars represent median values, boxes extend from the 25th to the 75th percentile, and whiskers represent the min to max range. *p* Values are derived from non-parametric Mann Whitney *U* tests. ****p ≤ 0.0001, ***p < 0.001, **p < 0.01, and *p < 0.05. Numbers of mice per group ranged from 10 to 13.

Thus far, our data suggest that calorie restriction attenuates age-associated effects in T cells but, conversely, accelerates the effects of aging in NK cells, and that the effect of calorie restriction is much more marked in C57BL/6 mice than in DBA/2 mice. To further investigate this unexpected effect of CR on NK cells, we analyzed expression of the maturation markers CD11b and CD27, which, together, define four stages of NK cell differentiation/maturation from Stage 1 (CD11b^−^ CD27^−^) to Stage 4 (CD11b^+^ CD27^−^) (Figure 5A) (41). Previous research indicates that accumulation of the most mature NK cell subset (Stage 4) is attenuated with increasing age (30, 44) and by CR (30, 44). In our study, however, the effect of age *per se* on the distribution of NK cell subsets was modest and restricted to a small—but statistically significant—increase in the proportion of Stage 2 cells (*p* = 0.014) and decrease in the proportion of Stage 4 cells (*p* = 0.02) with increasing age (Figure 5B). On the other hand and in agreement with the previous study (30, 44), we find that CR has a dramatic effect on NK maturation at all ages, increasing the proportions of the relatively immature Stage 2 subset and decreasing the proportions of the most mature Stage 3 and Stage 4 subsets, leading to marked differences in the ratio of Stage 2 to Stage 4 cells in CR mice compared to controls (Figure 5C). Amongst C57BL/6, but not DBA/2 mice, the ratio of Stage 2 to Stage 4 NK cells also increases with age (*p* = 0.036) and there is a significant interaction between age and diet (*p* = 0.005).

**Figure 6:**
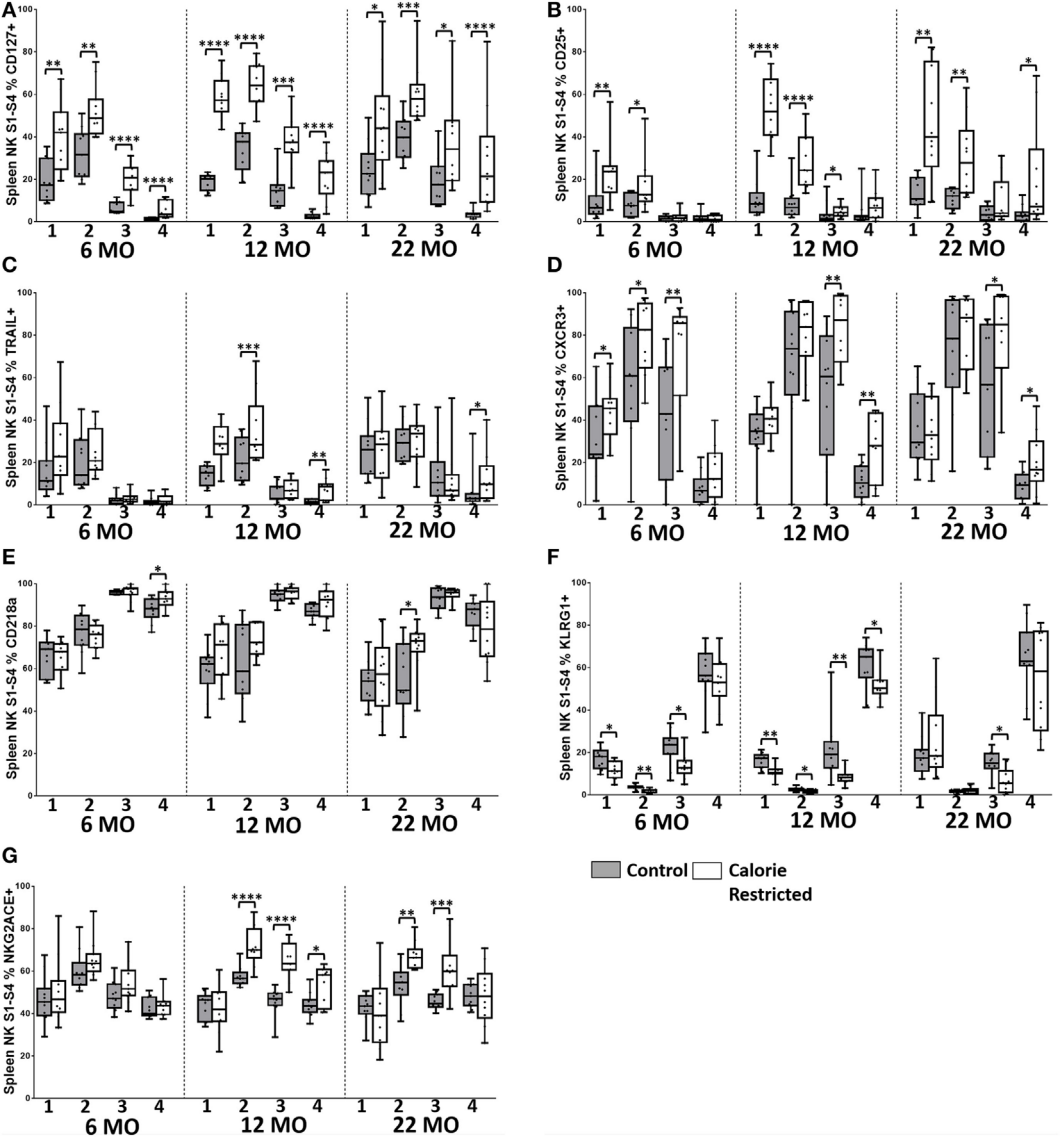
**Natural killer (NK) cell maturation status only partially explains altered NK cell phenotype in C57BL/6 calorie-restricted (CR) mice**. Characterization of C57BL/6 splenic NK cells by maturation status (numbered 1 through 4 below the *x*-axis, as defined in Figure 5A) in control (shaded boxes) and CR mice (open boxes) at 6 months (6 MO), 12 months (12 MO), and 22 months (22 MO) of age. **(A–G)** Plots represent the percentage of NK cells (NKp46^+^, CD3^−^) in each CD27/CD11b defined subset expressing **(A)** CD127, **(B)** CD25, **(C)** TRAIL, **(D)** CXCR3, **(E)** CD218a, **(F)** KLRG1, and **(G)** NKG2A/C/E. For gating, see Figure S4 in Supplementary Material. Horizontal bars represent median values, boxes extend from the 25th to the 75th percentile, and whiskers represent the min to max range. *p* Values are derived from non-parametric Mann Whitney *U* tests. ****p ≤ 0.0001, ***p < 0.001, **p < 0.01, and *p < 0.05. Numbers of mice per group ranged from 10 to 13.

**Figure 7:**
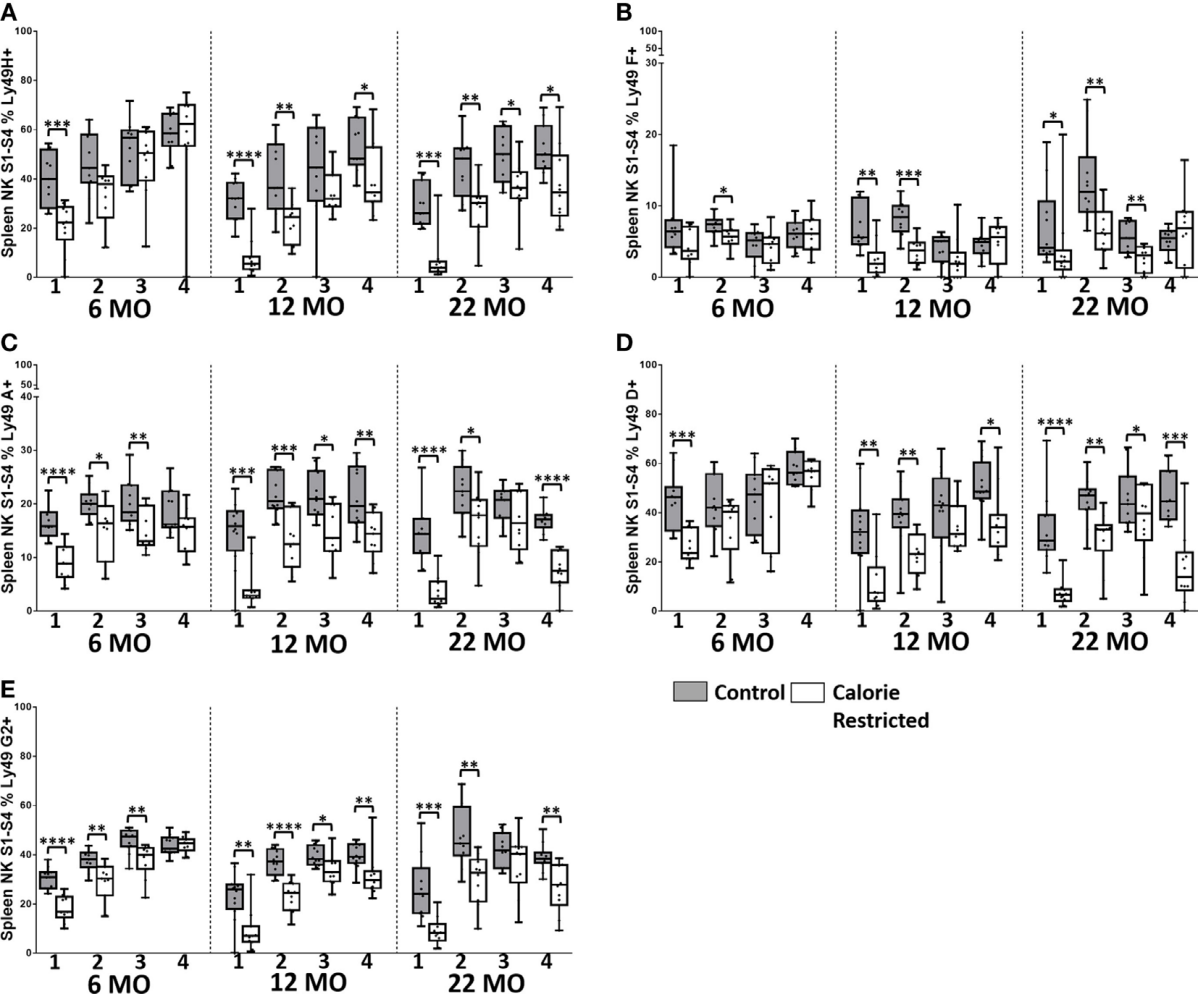
**Natural killer (NK) cell maturation status only partially explains altered LY49R expression in calorie-restricted (CR) mice**. Characterization of C57BL/6 murine splenic NK cells by maturation status (numbered 1 through 4 below the *x*-axis, as defined in Figure 5A) in control (shaded boxes) and CR mice (open boxes) at 6 months (6 MO), 12 months (12 MO), and 22 months (22 MO). **(A–E)** Plots represent the percentage NK cells (NKp46^+^, CD3^−^) in each CD27/CD11b defined subset expressing **(A)** Ly49H, **(B)** Ly49F, **(C)** Ly49A, **(D)** Ly49D, and **(E)** Ly49G2. For gating, see Figure S4 in Supplementary Material. Horizontal bars represent median values, boxes extend from the 25th to the 75th percentile, and whiskers represent the min to max range. *p* Values are derived from non-parametric Mann Whitney *U* tests. ****p ≤ 0.0001, ***p < 0.001, **p < 0.01, and *p < 0.05. Numbers of mice per group ranged from 10 to 13.

To determine whether this remarkable effect of calorie restriction on NK differentiation/maturation explains the differences in NK cell phenotypes described earlier (Figures 3 and 4), we compared expression of the various phenotypic markers between the Stages 1 and 4 subsets in C57BL/6 (Figures 6 and 7) and DBA/2 mice (Figure S5 in Supplementary Material).

Among C57BL/6 mice, CD127 (IL-7Rα), CD25 (IL-2Rα), and TRAIL were found to be more highly expressed on the least mature NK cells (Stage 1 and Stage 2) than on the more mature stages (Stage 3 and Stage 4) (Figures 6A–C). CXCR3 and NKG2A/C/E expression were highest on early to mid-stage NK cells (Stages 2–3) (Figures 6D,E) whereas KLRG1 and CD218a (IL-18Rα) (Figures 6F,G) and Ly49 receptors (Figure 7) were more frequently expressed by late stage NK cells. There were no significant changes in expression of these markers with increasing age in Stage 1 and Stage 2 NK cells, but proportions of Stage 3 and Stage 4 cells expressing CD127, CD25, and TRAIL did increase significantly with increasing age (*p* < 0.003 in all cases). However, there was a clear tendency for CR to enhance the expression of markers associated with immaturity (CD127, CD25, TRAIL, CXCR3, and NKG2A/C/E) and to decrease expression of markers associated with maturity (KLRG1, Ly49), on *all* NK cell subsets and there was a significant interaction between age and diet for expression of CD127 and CD25 on Stage 4 cells. In other words, CR not only delays the maturation of NK cells *through* the four CD11b/CD27-defined subsets but also delays the maturation of NK cells *within* each subset. This effect is, again, more marked for C57BL/6 mice than for DBA/2 mice although very similar trends were observed in both strains.

### Functional Effects of Calorie Restriction and Age

**Figure 8:**
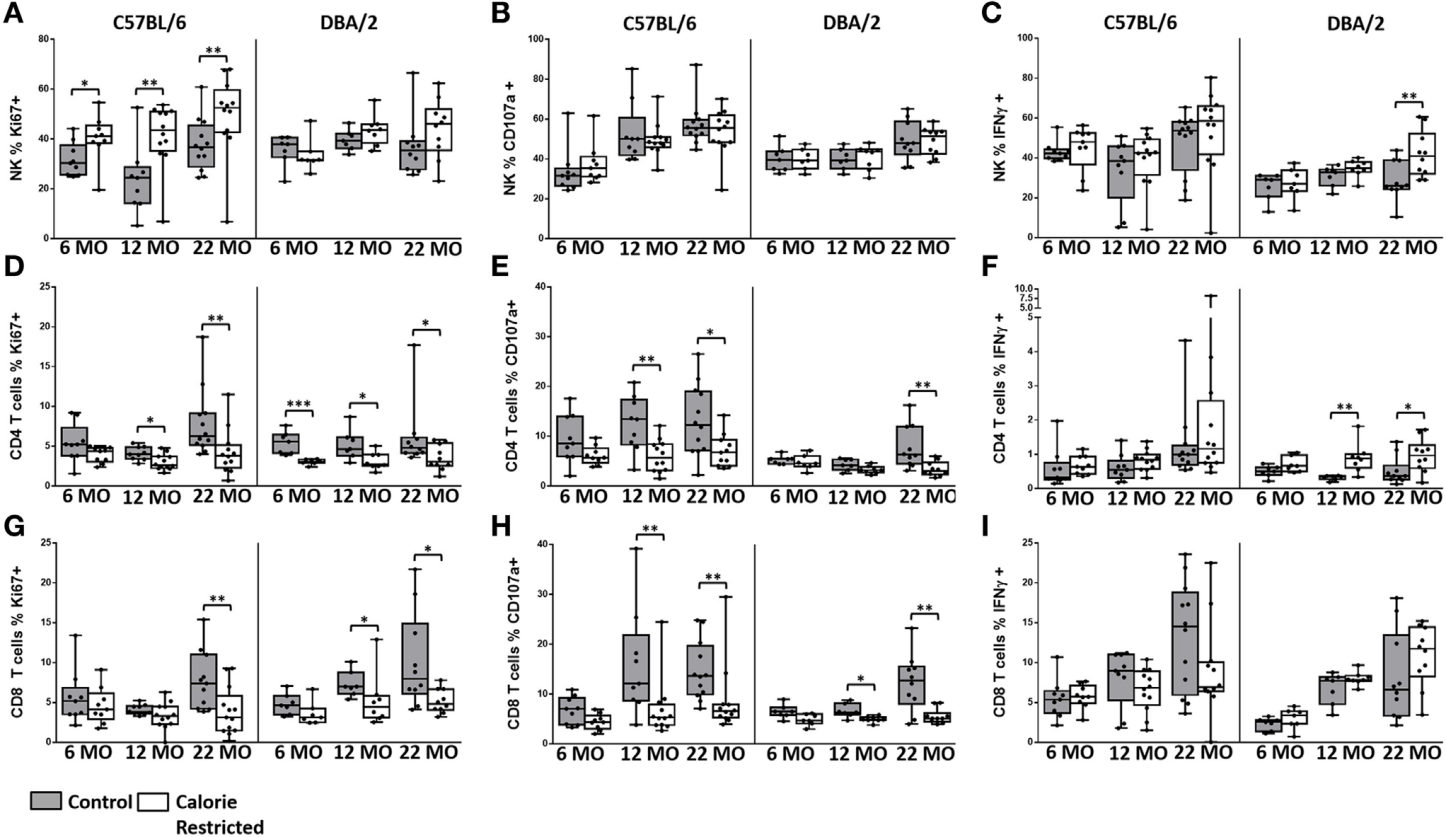
**Natural killer (NK) cell and T cell function are altered by age and calorie-restricted (CR)**. Characterization of murine splenic NK cells and T cells in control (shaded boxes) and CR mice (open boxes) at 6 months (6 MO), 12 months (12 MO), and 22 months (22 MO) of age in C57BL/6J and DBA/2J mice. Significant differences between strains are shown in parentheses. Splenic lymphocytes were stimulated for 18 h with IL-12 (5 ng/ml) and IL-18 (20 ng/ml) **(A–C,E,F,H,I)** or 100 ng/ml IL-2 **(D,G)**. **(A–C)** Percentage of NK cells (NKp46^+^ CD3^−^) expressing **(A)** Ki67 (12 MO C*), **(B)** CD107a (6 MO C*; 12 MO C*, CR**), and **(C)** IFNγ; **(D–F)** percentage of CD4^+^ T cells (CD3^+^ CD4^+^ or NKp46^−^) expressing **(D)** Ki67, **(E)** CD107a (6 MO C**; 12 MO C**; 22 MO C*, CR**), and **(F)** IFNγ (22 MO C**); **(G–I)** percentage of CD8^+^ T cells (CD3^+^ CD4^−^ NKp46^−^) expressing **(G)** Ki67 (12 MO CR***), **(H)** CD107a (12 MO C*), and **(I)** IFNγ (6 MO C**, CR**). For gating, see Figure S6 in Supplementary Material. Horizontal bars represent median values, boxes extend from the 25th to the 75th percentile, and whiskers represent the min to max range. *p* Values are derived from non-parametric Mann Whitney *U* tests. ****p ≤ 0.0001, ***p < 0.001, **p < 0.01, and *p < 0.05. Numbers of mice per group ranged from 10 to 13.

To begin to determine the functional consequences of these age- and nutrition-associated changes in T cell and NK cell phenotype, we cultured spleen cells *in vitro* with IL-12 plus IL-18 or with IL-2 for 18 h and examined them by flow cytometry for expression of the proliferation marker, Ki67, intracellular IFN-γ, and surface expression of CD107a expression (Figure 8; gating shown in Figure S6 in Supplementary Material).

Among C57BL/6 mice, there was a tendency for NK cell proliferative responses (Ki67) to IL-12/18 to increase with increasing age (*p* = 0.03) and to be higher among CR mice than among controls. NK cell degranulation (CD107a) responses and, to a lesser extent, IFN-γ responses also increased with age (*p* < 0.001 and *p* = 0.04, respectively) but there was remarkably little effect of CR on cytokine-induced NK cell degranulation or IFN-γ responses (Figures 8A–C). Again, the age-related effects were less pronounced in DBA/2 mice than in C57BL/6 mice (data not shown).

By contrast, although T cell proliferative CD107a and IFN-γ responses to IL-2 tended to increase slightly with age, cytokine-induced functional responses of both CD4^+^ and CD8^+^ T cells were significantly reduced among CR mice (Figures 8D–I). Expression of the activation marker CD69 increased with age on cytokine-activated NK cells and T cells but there was only a modest effect of calorie restriction (data not shown).

## Discussion

The conventional wisdom is that aging is associated with the accumulation of mature, terminally differentiated immune cells with restricted functional capacity, leading to loss of immune integrity (10, 14, 22, 23, 29, 30, 40–43, 47, 48, 54, 59), while calorie restriction is believed to preserve immune function, possibly by maintaining the pool of immune cell precursors or stem cells (4–6, 8–11). However, very few studies have looked at the effect of aging or calorie restriction on NK cell function and this is, to our knowledge, the first comprehensive study of the interaction between aging, nutritional status, genetic background, and NK cell and T cell phenotype and function.

Overall, calorie restriction had at least as big an effect as age on NK cell and T cell phenotype, and, where aging *per se* affected immune cells, these effects could be almost totally reversed (in the case of T cells) or were markedly exaggerated (in the case of NK cells) by calorie restriction. The very different effects of age and calorie restriction on T cell and NK cell differentiation and maturation suggest that, despite their many shared features, the underlying response of T cells and NK cells to increasing age and nutritional constraints is, mechanistically, very different.

Similarly, although the immunological effects of aging are very similar in C57BL/6 and DBA/2 mice, the effect of calorie restriction is much less obvious in DBA/2 mice than in C57BL/6 mice. This suggests, but does not prove, that the lack of benefit (in terms of longevity) from calorie restriction in DBA/2 mice (17, 18) may be associated in some way with the failure of the immune system to respond to the change in diet. It is known that DBA/2 mice have lower levels of oxidative stress, a higher metabolic rate, and reduced body fat acquisition with age, compared to C57BL/6 (16–19). Lower body fat acquisition in DBA/2 mice is not due to lower calorie intake in this strain or decreasing food intake with increasing age. Control DBA/2 mice, fed AL, ate (on average) 1–2 g more food each week than control C57BL/6 mice and their food intake remained constant over the lifespan; consequently, the calorie intake of CR DBA/2 mice was also marginally higher than that of CR C57BL/6 mice (*unpublished data; manuscript in preparation*).

The benefits of calorie restriction may thus be easier to observe in C57BL/6 mice as they are likely gain more from reduced body fat acquisition and oxidative stress with increasing age. Conversely, as DBA/2 mice require a higher energy intake to maintain a constant body weight and body fat (16, 18, 19), a 40% calorie restriction regimen may simply be too severe to see any overall benefit in this strain. This is reflected in our data. CD57BL/6 mice fed AL continued to gain weight throughout the lifespan and CR C57BL/6 mice were able to maintain a constant body weight as they aged, whereas DBA/2 mice fed AL maintained (but did not increase) their body weight [which is in agreement with previous studies (17, 19)] but were unable to maintain their body with increasing age when fed a CR diet. This loss of body mass with age under a 40% calorie restriction regimen may counteract any immunological benefit of calorie restriction leading to no overall increase in lifespan of DBA/2 mice. Repeating this study with a lesser degree of calorie restriction might begin to disentangle these issues.

As discussed above, an intriguing finding from this study is that calorie restriction potentiates age-associated changes in NK cell phenotype and function while simultaneously ameliorating age-associated changes in T cells. However, given the different age-related trajectories of T cell and NK cell populations in control animals, the overall effect of calorie restriction is to maintain larger populations of immature or less differentiated T cells and NK cells. Among NK cells, the maintenance of a less mature phenotype is reflected functionally with increased proliferative responses to cytokine stimulation. This is in partial agreement with evidence from mice (41, 60) and humans (32, 34, 35, 40), which indicates that less differentiated NK cells express high levels of cytokine receptors and respond strongly to cytokine-mediated signals whereas more mature NK cell subsets respond preferentially to cross-linking of natural cytotoxicity receptors by target cell surface ligands or cross-linking of CD16 by immune complexes (35–38, 40). Calorie restriction has been reported to increase the severity and/or mortality of influenza infection (14, 29, 42, 45, 61), West Nile virus infection (62), intestinal parasite infections (63), and autoimmune diseases (64) and this has been assumed to reflect loss of immune function. However, many of these diseases can in themselves cause rapid and substantial weight loss and it cannot be ruled out that this, rather than immune dysfunction *per se*, is the primary cause of increased mortality in animals that are already significantly underweight. The data presented here suggest, perhaps surprisingly given the extent of the changes in NK cell subset distribution, that calorie restriction has only a very limited effect on NK-cell degranulation and IFN-γ responses, although it is possible that testing NK cell function with a wider array of stimuli, including pathogens and MHC Class I deficient target cells may reveal other effects of calorie restriction. On the other hand, calorie restriction markedly reduced T cell proliferative and degranulation responses to IL-12/IL-18 stimulation but with much less effect on IFN-γ production. This may reflect reduced functional capacity at the level of individual T cells but might more easily be explained by underlying changes in the structure of the T cell population, particularly the very marked effect of calorie restriction on the accumulation of terminally differentiated CD8^+^ T cells as well as the tendency to preserve CD8^+^ T cells at the expense of CD4^+^ T cells, at least in C57BL/6 mice.

Overall, the effect of calorie restriction is that 22-month-old CR mice retain the immune cell phenotype of 6-month-old conventionally reared mice—the underlying basis for this is not entirely clear. Retention of an immature T cell and NK cell phenotypes in aged, CR mice may result from continuing production of new, immature cells; from accelerated turnover and apoptosis of mature cells; from extension of the life span of individual cells such that they take much longer to mature; or from a combination of any or all of these processes. It has been suggested that the reduced number of NK cells in aged mice is due to decreased trafficking out of the bone marrow (30, 60, 65). However, we observed a significant age-related decrease in the proportion of NK cells among splenic lymphocytes in DBA/2 mice but not in C57BL/6 mice and, although we do not have whole spleen cell counts and thus cannot calculate the exact number of NK cells per spleen, NK cell proportions were not affected by calorie intake suggesting that enhanced production of new NK cells from primary lymphoid organs is an unlikely explanation for our findings.

On the other hand, the markedly increased proliferative potential of NK cells from CR C57BL/6 mice suggests that early stage NK cells from CR mice may retain the potential for proliferative self-renewal: continual renewal of early stage (immature) NK cells would maintain these cells at a higher prevalence compared to later stage (more differentiated) subsets. This notion is supported by higher levels of expression of IL-7rα (CD127) *ex vivo* on NK cells from CR mice: IL-7 is a stromal cell-derived hematopoietic growth factor that is required for homeostatic proliferation and survival of NK cells (53, 66). However, previous observations suggest that CD127^+^ NK cell numbers are similar in control and CR mice at 6 months of age but that numbers of other NK cell subsets are lower (44). Taken together, these data suggest that premature loss of mature NK cell subsets in CR mice may lead to compensatory proliferation of immature subsets. This might also explain why NK cells from CR mice maintain a more immature phenotype irrespective of their CD11b/CD27-defined maturation stage: accelerated loss of mature NK cells may lead to more rapid differentiation of cells through the CD11b/CD27-defined stages but without the usual accompanying phenotypic and functional maturation.

Importantly, the effects of calorie restriction on lymphoid cell populations in lung, liver and lymph nodes were identical to those seen in the spleen (data not shown, but available on request) indicating that the effect of calorie-restriction is system-wide. It is not yet possible to say whether these immunological effects of calorie restriction in aging mice contribute to their enhanced life span but the correlation between the extent of the immunological changes and lifespan extension in C57BL/6 and DBA/2 mice is intriguing. Direct manipulation of the immune system in AL fed mice may provide more direct evidence to support or refute this theory. Just as importantly, we need to know whether similar effects are seen in humans. There are similarities between mice and humans in the immune consequences of aging (67, 68) but studies to assess the long-term effects of calorie restriction in humans are only just beginning (69). Our data suggest that calorie restriction may preserve immune function in later life but this is only likely to be beneficial if improved immune function can be achieved whilst still maintaining the energy reserves required to fight infection. More research is required to better understand the interaction between nutritional status, immune function, and healthy aging, in relevant animal models and in humans.

## Author Contributions

MW, CMB, MG, CN, A-SW, LB, CW, and JM conducted experiments, collected samples, collected and analyzed data, and contributed to writing of the manuscript. CB carried out statistical analysis of data. MW, DP, and ER designed experiments, interpreted data, and wrote the manuscript. DP and ER supervised research.

## Conflict of Interest Statement

The authors declare that the research was conducted in the absence of any commercial or financial relationships that could be construed as a potential conflict of interest.

## Abbreviations

ADCC, antibody-dependent cellular cytotoxicity; AL, *ad libitum*; CR, calorie-restricted; MFI, mean fluorescence intensity; 6 MO, 6 months old; 12 MO, 12 months old; 22 MO, 22 months old.
