# Supplementary figures and images for: Calorie Restriction Attenuates Terminal Differentiation of Immune Cells
Source: Front Immunol. 2017 Jan 12;7:667. doi: 10.3389/fimmu.2016.00667 (PMC5226962; doi:10.3389/fimmu.2016.00667)

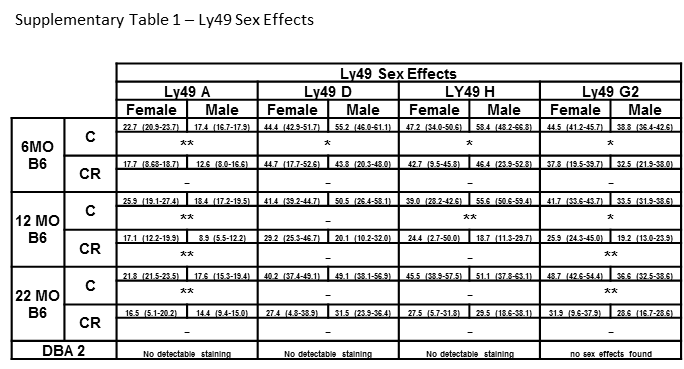

Supplement: Supplementary file 1 [file Table_1.DOCX]

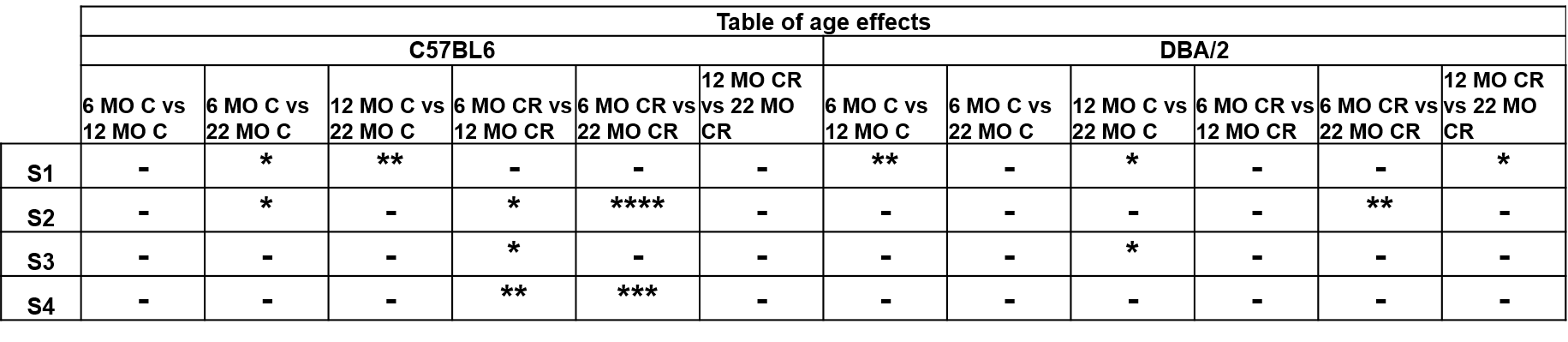


Supplementary Table 2 – NK cell subsets ageing effects

Supplement: Supplementary file 2 [file Table_2.DOCX]

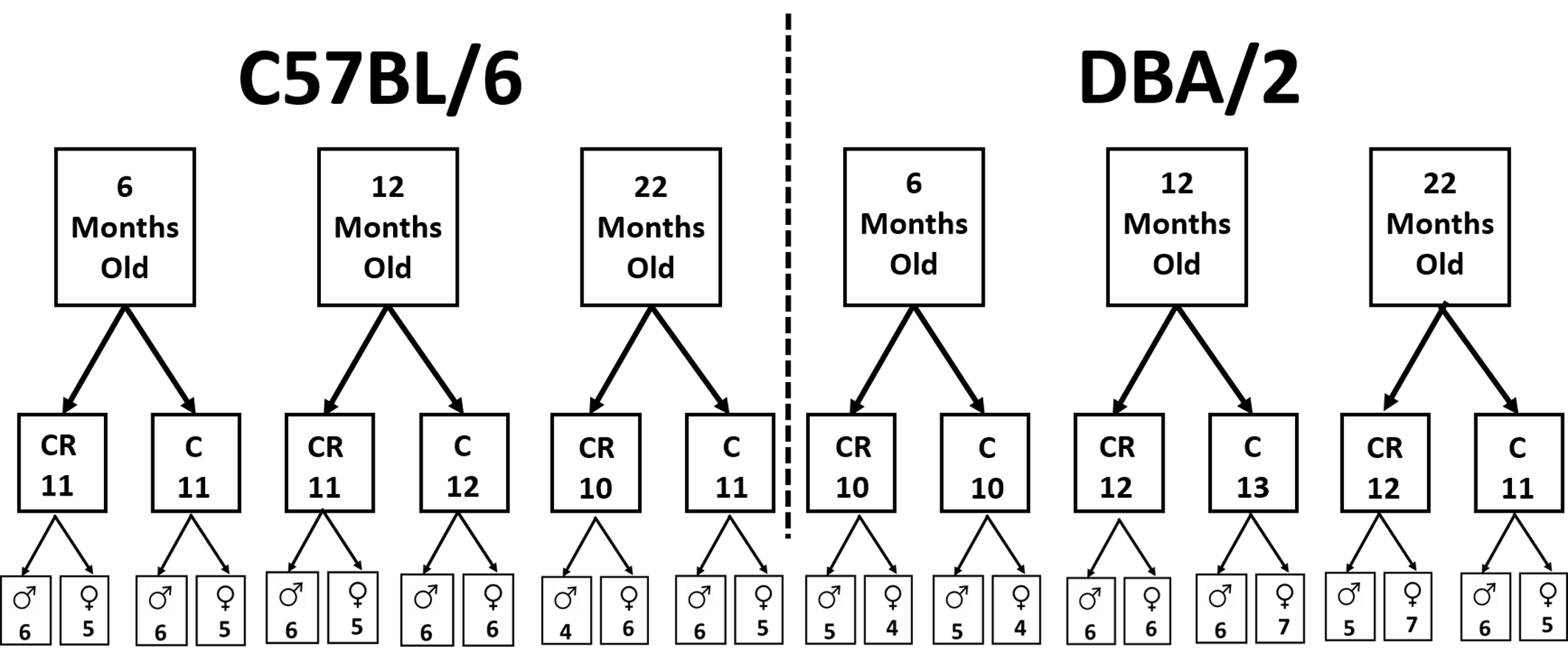

Supplement: Supplementary file 3 [file Image_1.TIF]

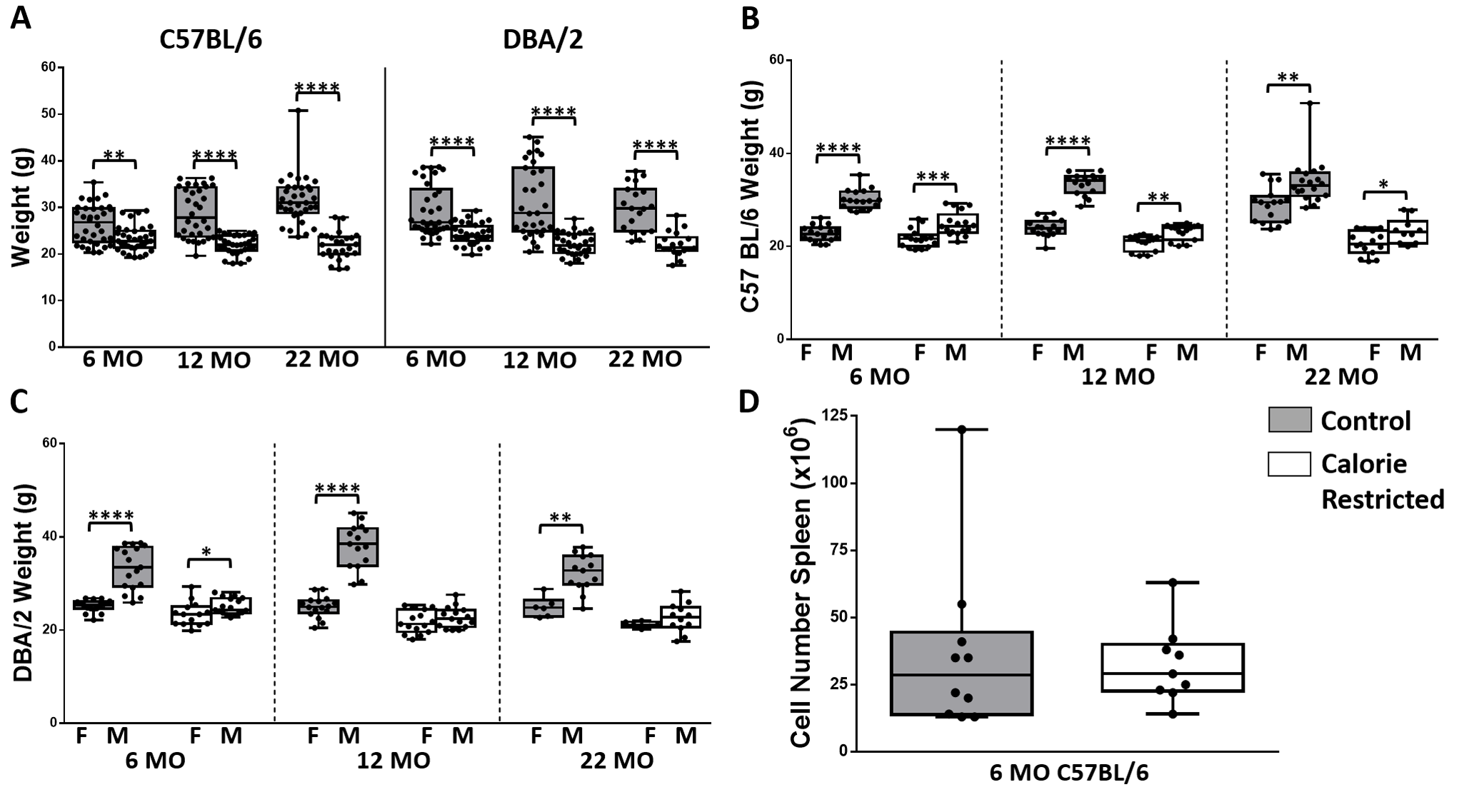

Supplement: Supplementary file 4 [file Image_2.TIF]

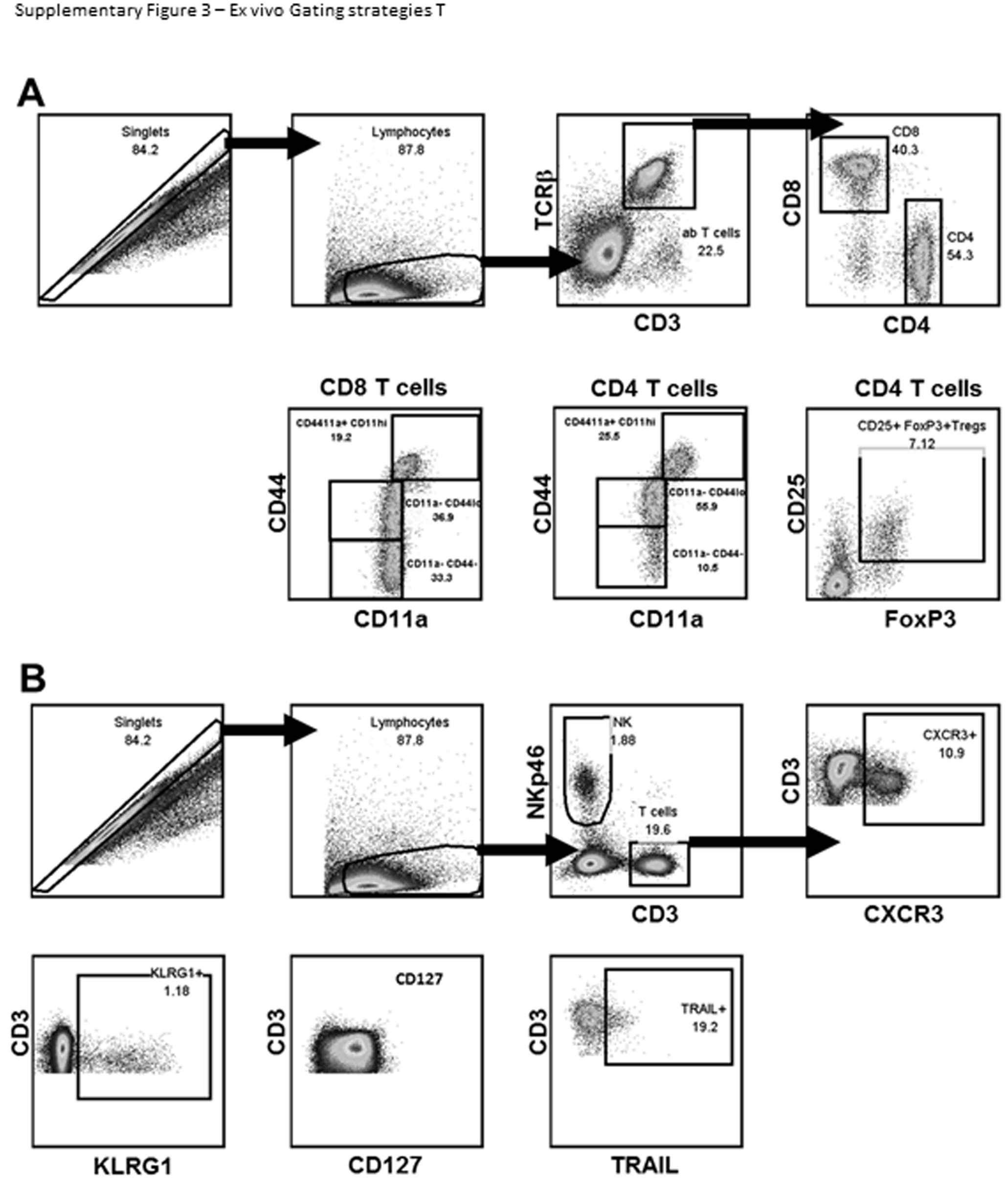

Supplement: Supplementary file 5 [file Image_3.TIF]

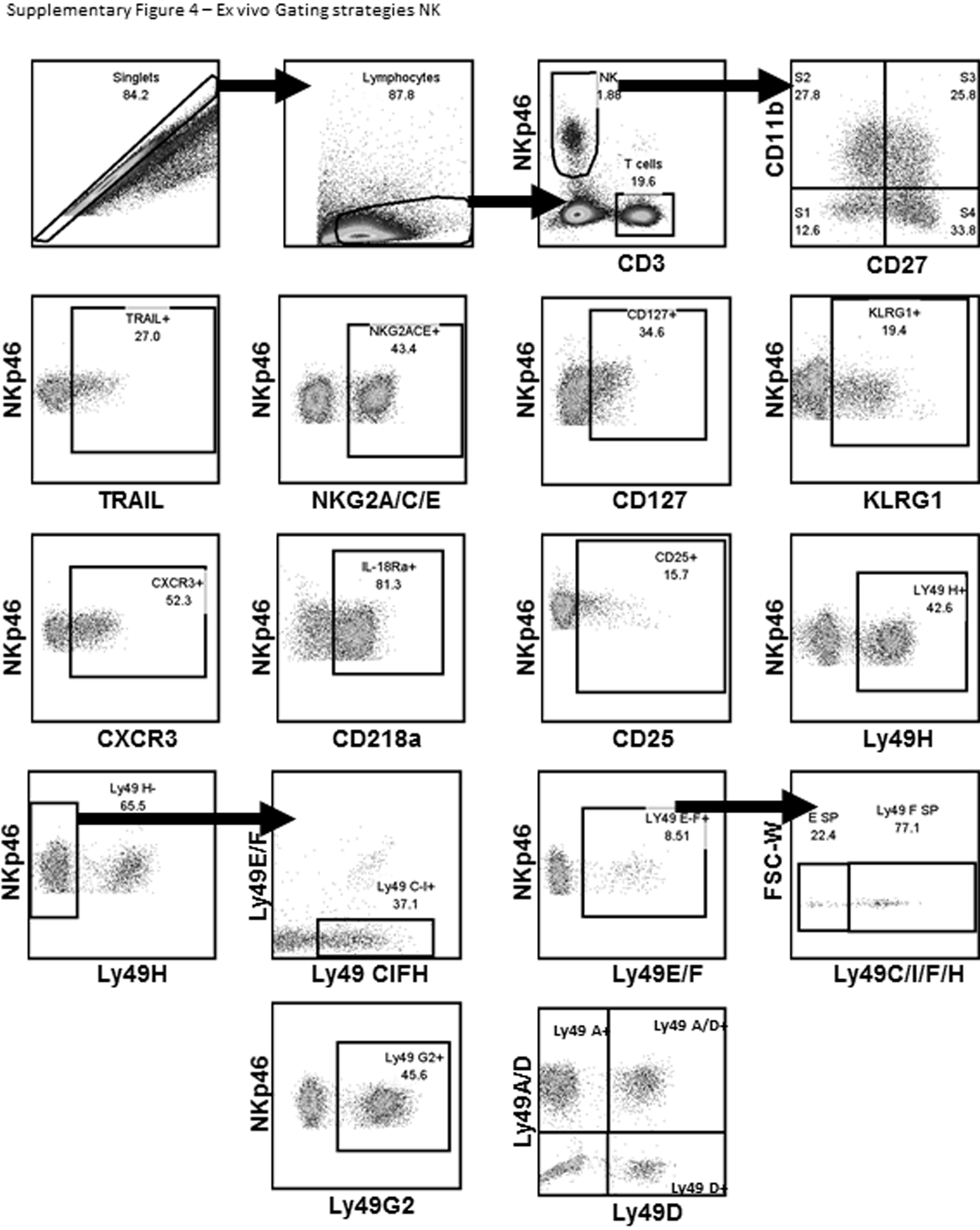

Supplement: Supplementary file 6 [file Image_4.TIF]

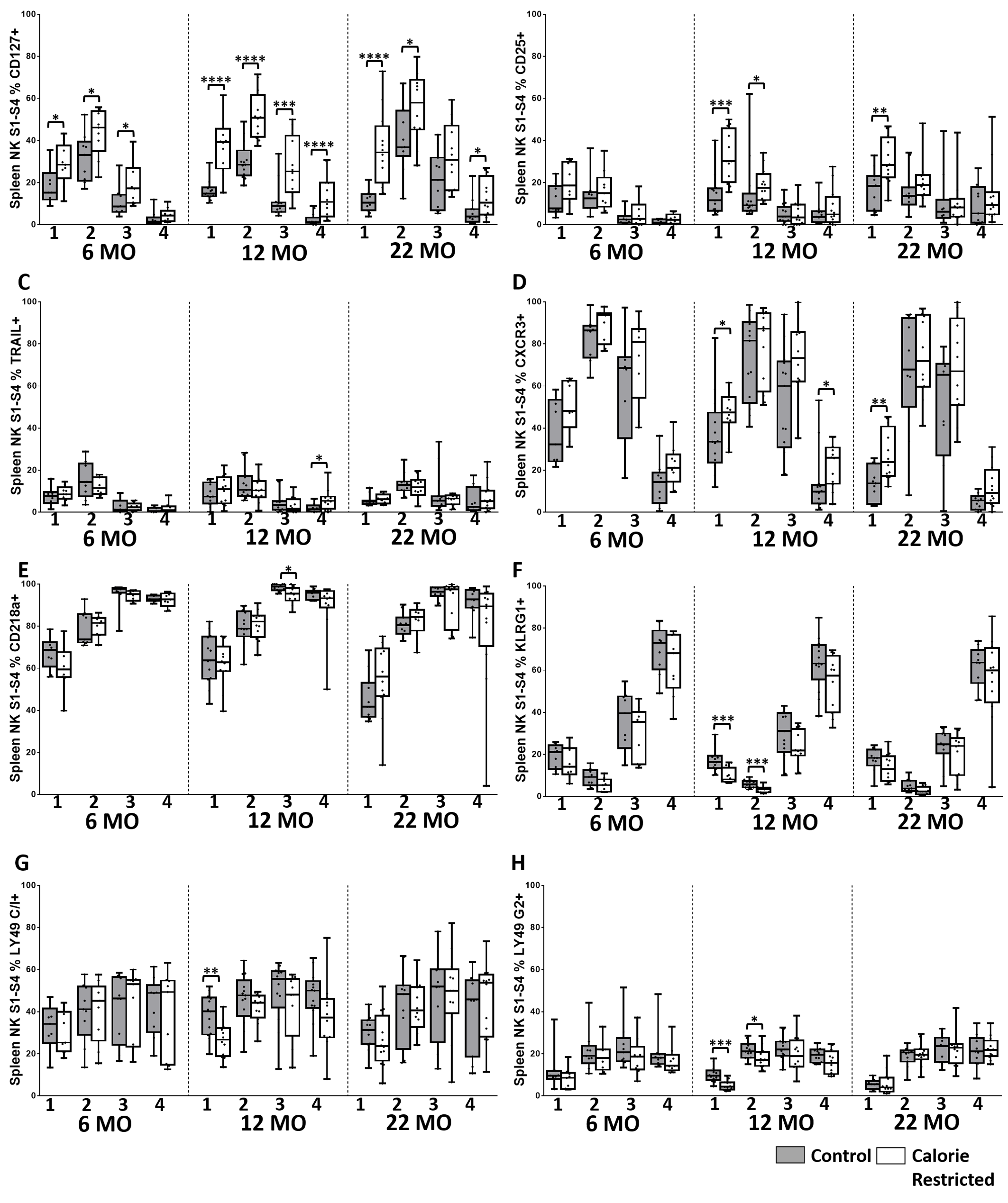

Supplement: Supplementary file 7 [file Image_5.TIF]

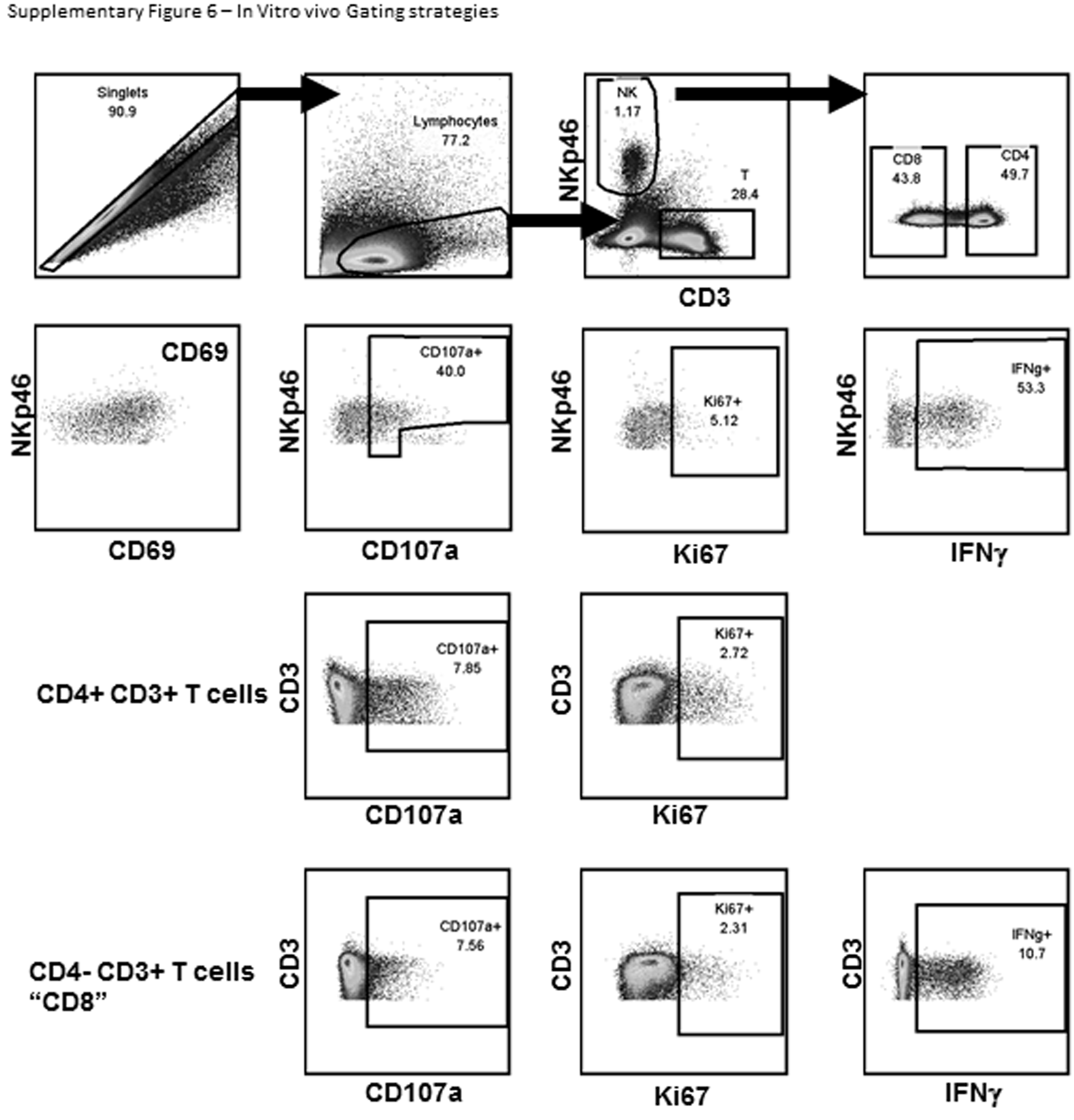

Supplement: Supplementary file 8 [file Image_6.TIF]
